# A randomized controlled trial of therapist-facilitated brief online behavioral parent training for reducing child disruptive behavior

**DOI:** 10.1016/j.ijchp.2024.100448

**Published:** 2024-02-09

**Authors:** Triet Pham, Dave Pasalich, Phu Tran, Richard O'Kearney

**Affiliations:** a School of Medicine and Psychology, ANU College Health and Medicine, The Australian National University, Building 39, Science Road, Canberra, ACT 2601, Australia; b Children's Hospital 1 (Benh vien Nhi dong 1), 341 Su Van Hanh street, District 10, Ho Chi Minh City, Viet Nam

**Keywords:** Disruptive behavior, Parent training, Online intervention, Brief intervention, Preschool children, Vietnam

## Abstract

**Background:**

Addressing child disruptive behavior in low and middle-income countries (LMICs) is challenging. Therapist-facilitated, multisession, brief, online group parent training offers hope for mitigating this issue. However, trials, particularly in Asia, are limited.

**Objective:**

This study primarily assessed the effectiveness of Brief Behavior Parent Training Vietnam (BBPTV) in reducing child disruptive behavior.

**Method:**

This study was a randomized controlled trial involving 109 Vietnamese parents (mean age = 34.1, 96 % were mothers) of preschool children displaying ongoing disruptive behaviors. Interventions included the BBPTV group (*n* = 56) receiving a therapist-facilitated, four-session program conducted through online group meetings and the care-as-usual (CAU) group (*n* = 53) having a 15 min individual online consultation. Primary outcomes, assessed online at two and six months postintervention, encompassed the intensity and frequency of children's disruptive problems. Secondary outcomes involved parenting practices, coercive interactions, marital conflicts, parenting self-efficacy, and parental mental health.

**Results:**

In contrast to CAU, the BBPTV group showed lower child disruptive intensity, reduced parent-child coercive interactions, and diminished marital conflicts, with a higher score in involving parenting two months post-intervention. Six months postintervention, BBPTV also exhibited significantly lower scores in child disruptive intensity and problems, harsh parenting, and coercive processes compared to CAU.

**Conclusions:**

The therapist-facilitated, four-session, internet-delivered group parent intervention resulted in superior and sustained improvements in child disruptive behavior, parenting practices, and parent-child coercive interaction compared to usual care, highlighting the potential for online BBPT to extend mental health care in Vietnam and other LMICs.

## Introduction

Disruptive behavior disorders are a prevalent mental health problem among children, with a global estimated prevalence rate of 8 % (Mohammadi et al., 2021). These disorders impact affected children and families, causing distress, impaired functioning (Loeber et al., 2009), and significantly rising social costs (Foster & Jones, 2005). Increased child disruptive behaviors relate to elevated harsh parenting practices (Lavigne et al., 2016), coercive family process (Lunkenheimer et al., 2016), parental mental health problems (Antúnez et al., 2018), marital conflicts (Cummings et al., 2004), and reduced parenting self-efficacy (Latham et al., 2018). Primary interventions and preventions target preschool children (Gardner et al., 2007).

Behavioral parent training (BPT) programs effectively reduce child disruptive behavior and improve relevant parenting factors (Kaminski & Claussen, 2017). However, standard BPT programs are constrained by lengthy duration and extensive training requirements for the trainers (Eyberg et al., 2008), leading to implementation challenges in low and middle-income countries (LMICs). Therefore, the development of brief multisession BPT (BBPT) programs has gained traction.

Evidence shows that BBPT interventions improve child disruptive behavior, parenting practices, parental stress, and parenting self-efficacy. These positive effects were observed in an Australian study employing three to four thirty-minute individual family sessions (Turner & Sanders, 2006), a Canadian trial on four two-hour group sessions (Bradley et al., 2003), and a three to five one-hour individual family intervention in Norway (Kjøbli & Bjørnebekk, 2013). The improvements were sustained for six months (Kjøbli & Bjørnebekk, 2013; Turner & Sanders, 2006) to one year post-intervention (Bradley et al., 2003).

Therapist-facilitated, psychological, internet-delivered intervention using video conference has been recommended to address limited healthcare resources (Comer et al., 2015). Recent trials demonstrate that internet-delivered, standard BPT programs achieve similar effects to clinic-based interventions in reducing child disruptive behavior (Comer et al., 2017; Dadds et al., 2019).

Given the evidence, therapist-facilitated, multisession, brief, online BPT group intervention (online BBPT) might effectively tackle mental health service gaps in LMICs. However, research primarily from high-income Western countries might limit the findings’ applicability to LMICs because sociocultural factors can affect parenting practices differently. Additionally, the online BBPT's effectiveness remains uncertain, as current evidence focuses on standard BPT programs. The data underscore the need for assessing online BBPT's impact, particularly in LMICs.

Vietnam, a Southeast Asian lower-middle-income country (World Bank, 2013), has a population of 96 million, including five million children aged three to six (General Statistics Office, 2020). Approximately 70 % of the population uses the Internet (Kemp, 2020). Confucianism strongly influences parenting, emphasizing strict filial obedience (Leung et al., 2009), resulting in parents' controlling, restrictive, and protective child-rearing methods (Guo, 2013). The prevalence of disruptive behavior among school-aged children is estimated at 8.7 % (Weiss et al., 2014). The country lacks adequately trained mental health professionals to address children's mental health problems (Cuong, 2017). Additionally, empirically psychological interventions explicitly developed for the country's context are limited (UNICEF Viet Nam, 2018). The evidence emphasizes the importance of researching online BBPT to tackle child disruptive behavior in Vietnam.

This study employed a randomized controlled trial (RCT) to assess the effectiveness of a four-session online BBPT in Vietnam, comparing it to the existing care option: online short consultations. The main hypothesis predicted that the intervention group parents would report a greater reduction in child disruptive behaviors than the control group. Secondary outcomes encompassed improvements in parenting practices, coercive family interaction, parental mental health, marital conflicts, and parenting self-confidence. Additionally, the study assessed the cultural acceptability, satisfaction, and parents' willingness to implement acquired parenting practices from the intervention.

## Methods

### Study design

We performed a two-parallel-arm RCT consistent with the Consolidated Standards of Reporting Trials (CONSORT) protocol, comparing online BBPT Vietnam (BBPTV; intervention) and Care as Usual (CAU; comparison). The study's protocol was approved by the University's Human Research Ethics Committee (HREC) and the Institutional Review Board of the Hospital (IRB). The trial was registered with the Australian New Zealand Clinical Trials Registry (ACTRN12620000176965).

All data were collected with voluntary written consent obtained online and were deidentified at baseline, one, two, and six months post-treatment. The recruitment was conducted online through three Vietnamese doctors’ Facebook pages from December 2020 to August 2021, with a follow-up completed in February 2022.

### Inclusion and exclusion criteria

Screening and recruitment were implemented using a self-report trial-developed screening questionnaire on the Qualtrics platform. The questionnaire included the Modified Checklist for Autism in Toddlers, Revised (MCHAT-R) (Robins et al., 2001), Pediatric Symptom Checklist-17 (PSC-17) (Jellinek & Murphy, 1990), Strength and Difficulties Questionnaire (SDQ) (Goodman, 1997), and Depression Anxiety Stress Scale 21 (DASS 21)(Lovibond & Lovibond, 1995).

The inclusion criteria for participants were: (1) children aged 36 to 72 months, with behavior problem scores ≥ 4 on the screening questionnaire derived from the SDQ and PSC-17′s externalizing behaviors, (2) native Vietnamese-speaking parents, (3) parental concerns regarding their child's behavior, and (4) availability of internet access.

Exclusion criteria for the trial included children with sensory/motor/language impairments, autism spectrum disorder (MCHAT-R score > 2), intellectual disability, mood disorders (PSC-17 internalizing score > 4), or those not living with parents. Additionally, parents receiving mental health treatment or experiencing any moderate level on any sub-scale of DASS 21 were excluded.

### Randomization

The primary investigator (PI) used software WINPEPI (Abramson, 2011) to create a pre-assessment list comprising 34 blocks with six randomized positions, aiming for a 1:1 allocation balance. After completing the baseline assessment questionnaire, participants were randomized and informed of their group assignment via email.

### Interventions

The manualized group intervention, BBPTV, conducted via Zoom, consisted of four 50 min sessions with up to 25 parents. Subsequent support was offered via telephone, email, and Facebook Messenger, if required, up to four instances within the eight-week post-intervention period.

The facilitators of BBPTV consisted of the PI and at least one assistant facilitator, who received training on behavioral modification and the BBPTV's content conducted by the PI. The assistant facilitators, medical doctors with at least two years of experience in the Psychology Department, moderated parents’ discussions.

BBPTV materials comprised a training manual, a PowerPoint presentation, and a fidelity checklist. The program imparted behavior modification techniques (see Table A.1 for details).

The CAU (control) was a 15 min individual Zoom consultation with the PI, using PowerPoint slides to deliver essential parenting practices (i.e., planned ignoring, time-out, understanding children's behaviors, and routinely playing). An information flyer was given afterward.

### Outcomes

Demographic information was provided by participants at enrolment, while other outcomes were assessed at baseline, one, two (primary time-point), and six months (follow-up) post-intervention. See Table A.2 for the psychometric data of the outcome measures in this study sample.

#### Primary outcomes

The outcomes were the intensity and frequency of child disruptive behavior. The 36-item Eyberg Child Behavior Inventory (ECBI) measured disruptive behavior intensity on a scale of 1 (never) to 7 (always) and behavior frequency with "yes" or "no" responses (Eyberg & Pincus, 1999).

#### Secondary outcomes

Secondary outcomes were the parenting practice dimensions measured by the Alabama Parenting Questionnaire (Frick, 1991) modified for preschool children – APQ (available on request). The measure comprised 27 items, assessing four parenting dimensions: Positive, Involving, Inconsistent, and Harsh dimensions; using a 1 (never) to 5 (always) scale. For cross-referencing, the trial used 21 questions from the Child Rearing Practices Questionnaire (CRPQ) (Atout et al., 2021) to examine Positive, Warmth, and Harsh parenting dimensions. Additionally, the nine-question Parent-Child Coercion Process Scale (PCCPS) (Mitnick et al., 2020) evaluated parent-child coercive interactions, both employing a frequency rating scale from 1 (never) to 5 (always).

The 16-item Parent Problems Checklist (PPC) (Stallman et al., 2009) evaluated marital conflicts, rating on a scale of 1 (not at all) to 7 (very much). The 17-question Parenting Sense of Competence Scale (PSOC)(Gibaud-Wallston & Wandersman, 2012) measured parenting self-efficacy, scoring from 1 (strongly disagree) to 6 (strongly agree). The 21-item DASS 21 (Lovibond & Lovibond, 1995) assessed parental mental health conditions, ranging from 0 (Did not apply to me at all) to 3 (Applied to me most of the time). Participants' acceptability, perceived usefulness, and likelihood of usage for each practice were rated from 1 (Not at all or never) to 5 (Absolutely acceptable/useful/likely use) after the final session of the BBPTV courses.

### Measures of harm

Participants exhibiting statistically significant increases in ECBI, APQ (harsh dimension), and DASS 21 scores at any post-assessment compared to baseline, as determined by the reliable change index (Jacobson & Truax, 1991), were subject to a deterioration protocol (available on request). Parents could also report concerns to the PI via email and Facebook Messenger. Any concerns regarding the study's conduct were emailed to the HREC, IRB, and the supervisor.

### Sample size

Due to the lack of previous research on online BBPT, the effect size of 0.45 on ECBI intensity from a clinic-based program was used (Kjøbli & Ogden, 2012). With ⍺ = 0.05, β = 0.2, the required sample size was determined as *n* = 78 per group, totaling 156 participants.

### Statistical analysis

The trial employed intention-to-treat analysis, with categorical variables presented as numbers and proportions and continuous variables as mean (SD). Repeated measures between-group ANOVA assessed outcome variables, considering the time (baseline, one, two, and six months post-intervention) and intervention type (BBPTV or CAU), except for attitudes towards BBPTV, which were analyzed descriptively. Missing data were imputed using SPSS Expectation-Maximization methods. Statistical significance was set at *p* < .05 (two-tailed). Data analysis employed SPSS statistical software version 26 and Microsoft Excel 365.

## Results

**Fig. 1:**
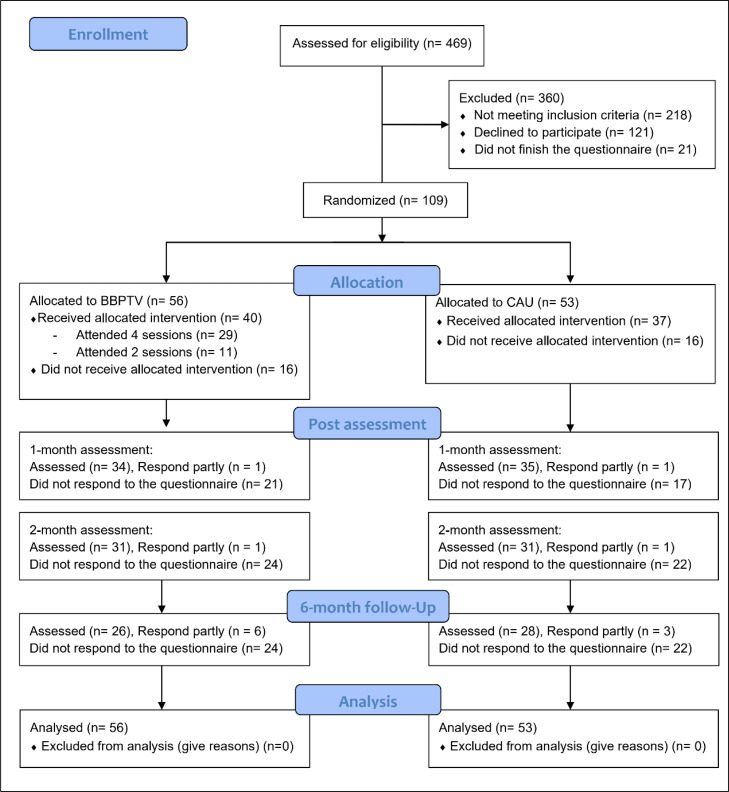
CONSORT participant's flow chart for the trial CAU Care as Usual, BBPTV Brief Behaviour Parent Training Viet.

Fig. 1 displays the CONSORT participant flowchart. About 29 % of participants received no intervention. Attrition rates were 34.9 %, 42.2 %, and 42.2 % at one, two, and six months post-intervention. Comparing non-dropout and dropout groups, at preintervention, participants in provinces (17/41) showed higher dropout likelihood than those in cities (15/68), *X^2^* (1, *n* = 109) = 4.64, *p* = .031. At two months, fathers (2/2) had higher dropout rates than mothers (14/75), *X^2^* (1, *n* = 77) = 7.83, *p* = .041.

The study involved 109 parents (mean age = 34.1, SD = 4.3); 96 % were mothers. Among the target children (mean age = 48.3, SD = 9.6 months), 69 (63.3 %) were male. No significant baseline differences between intervention groups were found (Tables 1 and 2).

**Table 1:** Baseline characteristics by intervention group.

Variable	CAU (*n* = 53)		BBPTV (*n* = 56)	
	M	SD	M	SD
Child's age (months)	47.45	9.98	49.16	9.42
Parent's age (years)	34.04	4.55	34.16	4.04
Number of children/ family	1.72	.60	1.78	.65
	n	%	n	%
Child's gender				
Male	38	71.7	31	55.4
Female	15	28.3	25	46.6
Child's order				
First	40	75.5	43	76.8
Second or third	13	24.5	13	23.2
Parent's gender				
Female	50	94.3	55	98.2
Male	3	5.7	1	1.8
Marital status				
Married	50	94.3	54	96.4
Others	3	5.7	2	3.6
Ethnic group				
Kinh	53	100	54	96.4
Others	0	0	2	.3.6
Education level				
College and below	4	7.5	5	8.9
Undergraduate and above	49	92.4	51	91.0
Job-status				
Having a job	45	84.9	52	92.9
Unemployed	8	15.1	4	7.1
Address				
Ho Chi Minh city/Hanoi	35	66.0	33	58.9
Other provinces	18	34.0	23	41.1
Living area				
Urban	48	90.6	46	82.1
Suburban	5	9.4	10	17.9

**Table 2:** Outcomes' mean scores at baseline, 2, and 6 months postintervention by intervention group.

	CAU (*n* = 53)						BBPTV (*n* = 56)						
	Baseline		Two months		Six months		Baseline		Two months		Six months		*p*^a^
Measures	M	SD	M	SD	M	SD	M	SD	M	SD	M	SD	
ECBI intensity	121.90	26.43	118.84	26.10	117.24	27.91	123.99	23.92	108.68	25.42	102.08	32.24	.66
ECBI problem	21.00	8.70	16.96	8.71	17.29	10.12	20.68	10.38	15.45	10.12	12.37	12.75	.86
APQ-Positive P.	30.92	3.54	33.45	3.77	34.08	3.37	30.83	2.82	32.25	3.44	33.18	4.22	.87
APQ-Involving P.	28.30	4.36	29.43	2.73	31.25	3.69	28.50	4.46	31.21	2.13	32.69	6.43	.81
APQ-Inconsistent P.	16.51	2.31	16.43	2.20	15.93	2.18	16.43	2.54	15.55	2.75	15.86	3.28	.86
APQ-Harsh P.	9.64	2.20	9.40	2.24	9.15	3.18	9.96	2.70	8.90	1.98	7.66	4.56	.49
CRPQ-Warmth P.	30.01	2.94	29.72	2.34	30.07	2.70	29.60	3.50	29.77	2.81	30.84	3.30	.51
CRPQ-Positive P.	32.03	3.36	33.15	3.76	33.21	3.42	30.88	3.71	32.81	4.31	32.53	3.67	.09
CRPQ-Harsh P.	12.53	3.10	12.04	3.07	11.60	3.80	13.14	3.55	11.46	3.07	10.30	5.44	.34
PCCPS	19.38	6.71	19.44	6.02	18.03	4.96	19.19	5.49	15.99	3.90	14.61	4.53	.85
PSOC	64.84	7.80	67.15	7.98	66.27	10.03	63.73	8.09	67.96	9.70	67.96	9.79	.47
PPC	42.28	13.06	40.71	13.68	37.48	10.49	37.81	10.10	32.84	12.63	36.54	14.08	.05
DASS	19.74	12.88	17.55	13.54	17.12	14.16	22.18	12.58	18.10	16.32	16.14	21.09	.32

CAU Care As Usual, BBPTV Brief Behaviour Parent Training Viet, ECBI Eyberg Child Behavior Inventory, APQ Alabama Parenting Questionnaire, CRPQ Child Rearing Practices Questionnaire, P. Parenting Practices, PCCPS Parent Child Coercive Process Scale. PSOC Parenting Sense Of Competence scale, PPC Parent Problem Checklist, DASS Depression Anxiety Stress Scale.

a Baseline comparison.

The ANOVA results showed significant interactions between intervention type and time across measures: ECBI intensity and problem, APQ and CRPQ harsh parenting, PCCPS, PSOC, PPC, and DASS, indicating the intervention's effects depending on time. Therefore, simple effect tests were performed to assess the interventions’ main effects on these measures (IBM Support, 2020).

**Fig. 2:**
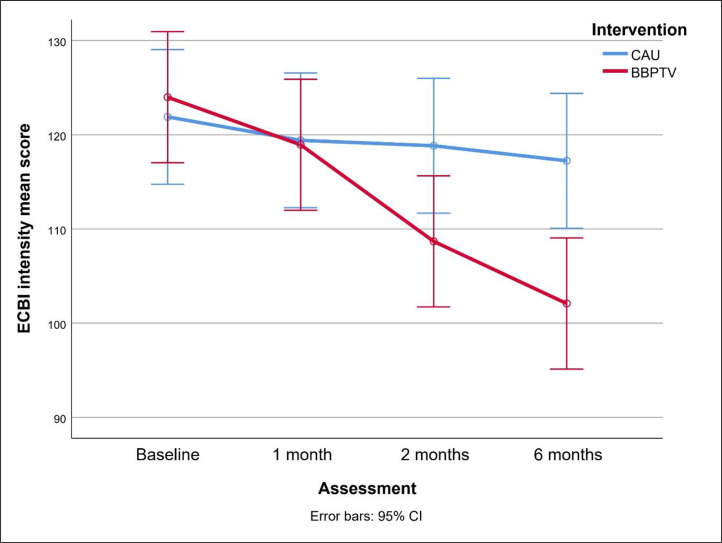
Changes in ECBI intensity means between BBPTV and CAU interventions Color should be used. ECBI Eyberg Child Behavior Inventory, CAU Care as Usual, BBPTV Brief Behaviour Parent Training Viet.

**Fig. 3:**
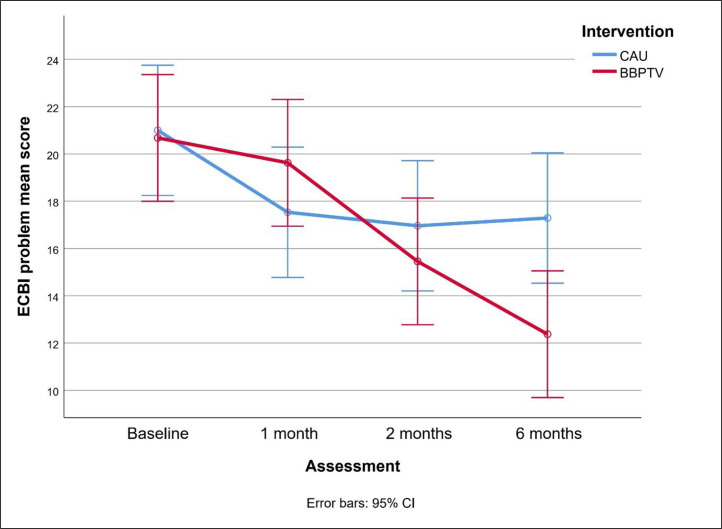
Changes in ECBI problem means between BBPTV and CAU interventions Color should be used. ECBI Eyberg Child Behavior Inventory, CAU Care as Usual, BBPTV Brief Behaviour Parent Training Viet.

Fig. 2, Fig. 3 compare changes in ECBI scores between BBPTV and CAU over the trial. BBPTV showed significantly lower ECBI intensity means at two-month [mean difference (95 % CI) = -10.15 (-20.13 to -0.17), *p* = .046, Cohen's d (95 % CI) = -0.39 (-0.78 to -0.02)] and six-month post-intervention assessments [mean difference = -15.15 (-25.14 to -5.17), *p* = .003, *d* = -0.50 (-0.89 to -0.12)]. At two months, ECBI problem means were slightly lower [mean difference = -1.51 (-5.10 to 2.08), *p* = .44, *d* = -0.16 (-0.54 to 0.22)], but significantly so at six months post-intervention [mean difference = -4.92 (-9.31 to -0.53), *p* = .012, *d* = -0.42 (-0.82 to -0.05)], favoring BBPTV.

Regarding secondary outcomes, at the two-month post-intervention mark, BBPTV parents reported significantly higher APQ-involving parenting and lower PCCPS and PPC scores than CAU parents, with no significant differences in other measures. At six months, BBPTV parents had significantly lower APQ-harsh parenting and PCCPS scores than CAU parents, with no differences in other measures (Table 3).

**Table 3:** Treatment comparisons on secondary outcomes by intervention group.

	CAU (control) vs. BBPTV (intervention)					
	Two months post-intervention			Six months post-intervention		
Measures	Mean Diff. (95 % CI)	*p*	Effect size (95 % CI)	Mean diff. (95 % Cl)	*p*	Effect size (95 % CI)
APQ-Positive P.	-1.20 (-2.57 to 0.17)	.06	-0.33 (-0.72 to 0.05)	-0.90 (-2.36 to 0.55)	.17	-0.23 (-0.62 to 0.14)
APQ-Involving P.	1.78 (0.85 to 2.71)	.023	.72 (0.35 to 1.13)	1.44 (-0.57 to 3.45)	.07	.27 (-0.11 to 0.66)
APQ-Inconsistent P.	-0.88 (-1.83 to 0.07)	.09	-0.35 (-0.74 to 0.03)	-0.07 (-1.13 to 0.99)	.90	-0.02 (-0.41 to 0.35)
APQ-Harsh P.^a^	-0.50 (-1.30 to 0.30)	.37	-0.24 (-0.62 to 0.14)	-1.49 (-2.99 to 0.01)	.007	-0.37 (-0.77 to 0.00)
CRPQ-Warmth P.	.05 (-0.93 to 1.03)	.93	.02 (-0.36 to 0.40)	.77 (-0.38 to 1.92)	.18	.25 (-0.12 to 0.64)
CRPQ-Positive P.	-0.34 (-1.88 to 1.20)	.63	-0.08 (-0.47 to 0.30)	-0.68 (-2.03 to 0.67)	.34	-0.19 (-0.57 to 0.19)
CRPQ-Harsh P.^a^	-0.58 (-1.75 to 0.59)	.40	.19 (-0.57 to 0.19)	-1.30 (-3.09 to 0.49)	.06	-0.27 (-0.66 to 0.10)
PCCPS^a^	-3.45 (-5.37 to -1.53)	.001	-0.68 (-1.08 to -0.30)	-3.42 (-5.22 to -1.62)	.001	-0.72 (-1.12 to -0.34)
PSOC^a^	.81 (-2.57 to 4.19)	.62	.09 (-0.29 to 0.47)	1.69 (-2.07 to 5.45)	.30	.17 (-0.21 to 0.55)
PPC^a^	-7.87 (-12.9 to -2.87)	.002	-0.59 (-0.99 to -0.22)	-0.94 (-5.68 to 3.80)	.70	-0.07 (-0.46 to 0.30)
DASS^a^	.55 (-5.16 to 6.26)	.85	.04 (-0.34 to 0.42)	-0.98 (-7.84 to 5.88)	.73	-0.05 (-0.43 to 0.32)

CAU Care As Usual, BBPTV Brief Behaviour Parent Training Viet, APQ Alabama Parenting Questionnaire, CRPQ Child Rearing Practices Questionnaire, P. Parenting Practice Dimension, PCCPS Parent Child Coercive Process Scale. PSOC Parenting Sense Of Competence scale, PPC Parent Problem Checklist, DASS Depression Anxiety Stress Scale. ^a^ Determined by simple effect test.

Among 61 participants in the BBPTV and CAU intervention groups (40 and 21, respectively) attending BBPTV classes, 37 voluntarily shared their opinions its parenting strategies. About 85 % of parents found the strategies acceptable and useful and showed intention to use them. However, around 10 to 15 % of participants expressed reservations concerning the effectiveness and acceptance of specific techniques such as time-out, purposely ignoring, problem-solving, effective command, and tangible rewards, reporting a lower likelihood of regular future utilization of these techniques.

Twenty-six out of 27 items on the BBPTV fidelity checklist were faithfully implemented, except for the facilitators' modeling of parenting strategies, which was specific to the in-person training. Though no checklist existed for short consultations, all flyer information was thoroughly explained. No significant adverse effects relating to the interventions were found during the trial.

## Discussion

Both interventions were associated with improvements in many outcomes (Table 2), contributing to the literature supporting the efficacy of online BBPT in reducing child disruptive behavior. The hypothesis that BBPTV would outperform CAU in improving measures of child disruptive behaviors all assessments was generally supported, aligning with the primary objective.

The BBPTV exhibited superior effectiveness over the CAU in reducing child disruptive behavior intensity after two months. Six months post-intervention, the BBPTV showed significantly better outcomes in decreasing the intensity and number of disruptive problems.

Nonetheless, the trial failed to establish the superiority of BBPTV over CAU in improving positive parenting practices, parenting self-efficacy, and parental mental health conditions. This outcome aligns with prior research that observed significant differences solely in child behaviors and negative parenting practices across intervention types (Sanders et al., 2000).

A plausible explanation is that, despite no difference in the frequency of positive parenting practices between the two groups, the BBPTV intervention enhanced the quality of these strategies better than CAU. Evidence indicates that parenting strategies' effectiveness in reducing child disruptive behaviors can be undermined due to improper implementation (Riley et al., 2017). By optimizing positive practice implementation, the BBPTV group significantly reduced child disruptive behavior more than the CAU.

Another possible explanation is that the BBPTV reduced harsh parenting practices more than CAU, resulting in a superior decrease in child disruptive behavior. Less harsh practices disrupt the coercive cycle, fostering positive communication and behaviors, and reducing disruptive problems. The study confirms this hypothesis by showing BBPTV's greater impact in reducing coercive processes.

The trial's final goal was to evaluate parents' perceptions of the BBPTV's techniques. As anticipated, most parents found the practices acceptable and useful, expressing their willingness to use them. However, a small proportion of participants showed uncertainty about the usefulness and likelihood of employing certain strategies, consistent with prior Vietnamese research (Tran & Weiss, 2015).

The influence of Confucianism, emphasizing strict child obedience (Leung et al., 2009), may lead Vietnamese parents to prioritize correcting undesirable behaviors through orders and advice rather than considering "purposely ignoring" and "problem-solving" methods as effective. Additionally, the popular saying "Thuong cho roi cho vot" (similar to “Spare the rod, spoil the child”) (Beazley, 2006) could discourage the use of non-physical discipline like "time-out." Moreover, misapplication of "time-out" might contribute to its perceived ineffectiveness (Riley et al., 2017).

The trial experienced attrition rates higher than expected but consistent with existing literature. Reviews indicate that attrition rates of online intervention vary from 18.8 % to 30.6 % pre-intervention and 22.5 % to 48.3 % during the intervention (Fernandez et al., 2015). The Covid pandemic context might contribute to the high attrition of the present study, as urgent concerns overshadowed child behavior problems.

While high attrition introduces potential bias and limits generalizability, it enables simultaneous intervention delivery to numerous parents. Moreover, this challenge can be mitigated through the cost-effectiveness of BBPTV. Unlike the CAU approach, in which a physician dedicates 15 min per parent, the BBPTV intervention requires only 8 min per parent in group training sessions if conducted by a single physician. This reduction in time is coupled with augmented dissemination of information and increased opportunities for parents to engage in practical exercises.

Some limitations should be acknowledged in this study. Firstly, the trial exclusively took place online, leading to a distinct participant demographic. For example, 90 % of participants held an undergraduate degree or higher, while the corresponding national rate in Vietnam is approximately 9.5 % (General Statistics Office, 2020). The difference may limit the generalizability of the findings to the broader population.

The second limitation is the reliance on a single informant assessment, potentially introducing biases in the gathered information. To enhance objectivity, future research should involve additional informants like fathers and teachers.

A third limitation is excluding families with limited internet access from receiving the intervention. Nonetheless, given that approximately 70 % of the Vietnamese population uses the internet (Kemp, 2020), with a significant portion at parental ages (Nguyen, 2020), the intervention holds promise for benefiting most of the community and healthcare system by mitigating the burden of mental health issues.

Fourth, time constraints led to a sample size below protocol requirements, potentially hindering result accuracy. Additionally, evaluations on BBPTV participant's views were limited; future research with focus group discussions is essential for a more comprehensive investigation.Fifth, due to the presence of numerous measures, the consideration of multiple significance testing as a limitation is warranted. Finally, the dual role of the PI in implementing both interventions and analyzing data may introduce bias. However, the study's high fidelity levels could minimize this bias.

## Conclusion

The study shows that a therapist-facilitated, four-session, online group parent intervention can effectively decrease disruptive behaviors in preschool children in Vietnam. The approach offers an alternative option to current practice for families who face barriers such as financial difficulties, distance, and social stigma preventing them from visiting a clinic in person. The brief online intervention is also a practical solution to Vietnam's mental health care situation. To our knowledge, the present study is the first trial to investigate the effects of a therapist-facilitated, multisession, brief, internet-delivered group parent training course for reducing child disruptive behavior in Asian LMICs. These findings provide an important opportunity to advance the understanding of transporting evidence-based psychological practices to new contexts and, therefore, be of value to researchers wishing to implement similar studies in low-resource areas.

## Financial disclosure

The study received financial support from the research fund of the ANU School of Medicine and Psychology, as well as the Australian Psychological Society Grant for Intercultural and/or International Projects (Year: 2019/2020).

## Declaration of competing interest

The authors declare that they have no known competing financial interests or personal relationships that could have appeared to influence the work reported in this paper.

## Figures and Tables

**Table A.1:** Fidelity checklist of the BBPTV program.

	Time (min.)	Check when completed
Session 1		
1. Introduction of facilitators	3	
2. Introduction of the training course: goals, schedule	1	
Introduction of Behavioral Theory		
3. Definition of a behavior, un- and desirable behavior	3	
4. Explaining the behavior theory – ABC	5	
Identifying functions of a behavior		
5. Introduction of reinforcement and its types	2	
6. Social reinforcement	2	
7. Tangible/activity reinforcement	2	
8. Sensory reinforcement	2	
Choosing Solutions		
9. Antecedent modification	2	
10. Consequence modification	2	
11. Group discussion: item 2,3,4	25	
Session 2		
Pragmatic Practices		
12. Special time:	2	
13. Child-led play.	2	
14. Parent-led play.	2	
Differential Reinforcement		
15. Positive attention to desirable behaviors: descriptive praise, physical affection, tangible reward.	4	
16. Negative attention to misbehaviors: Planned ignoring.	2	
17. Problem solving	2	
18. Effective command	2	
19. Time-out	4	
Practice Parenting Practices		
20. Facilitator modelling	5	
21. Parents practicing	20	
22. Homework and wrap -up of session 1 & 2	10	
Session 3		
23. Discussion on issues that cause difficult to participants when they conduct trained practices at home (homework)	20	
24. Dealing with the 3 most common child disruptive behaviors (group discussion)	30	
Session 4		
25. Practice parenting practices	30	
26. Participants report their attitude towards acceptability, perceived effectiveness, and prospective use of trained practices (Optional)	10	
27. Wrap-up of the course	5	

**Table A.2:** Summary of measures, number of items, and Cronbach alphas.

Measures	Number of items	Cronbach alpha
Primary outcomes		
ECBI intensity	36	.92
ECBI problem	36	.94
Secondary outcomes		
APQ-Positive P.	9	.54
APQ-Involving P.	8	.70
APQ-Inconsistent P.	6	.60
APQ-Harsh P.	4	.71
CRPQ-Positive P.	9	.67
CRPQ-Warmth P.	7	.80
CRPQ-Harsh P.	5	.79
PCCPS	9	.82
PSOC	17	.63
PPC	16	.86
DASS	21	.87

ECBI Eyberg Child Behavior Inventory, APQ Alabama Parenting Questionnaire, CRPQ Child Rearing Practices Questionnaire, P. Parenting Practices, PCCPS Parent Child Coercive Process Scale. PSOC Parenting Sense Of Competence scale, PPC Parent Problem Checklist, DASS Depression Anxiety Stress Scale.

## Appendix A

**Deterioration protocol**

**Step 1:** The mean score of any of measures ECBI, Harsh practices of APQ, and DASS of any parent-child dyad measured at time 2, 3, and 4 is statistically higher than those of the assessment 1 based on reliable change index.
**Step 2:** The primary investigator or assistant researcher contacts the participant as soon as possible to inform about the mean score(s) via telephone or Facebook messenger. Following is an example:
- *Good morning, my name's X. I am a researcher in the study* [name of the study] *that you are participating in. How are you today?*
- *I am monitoring the result of the assessment* [number of the assessment] *and find that the condition of your child problem behaviors/your mental health condition/the parenting practices does not show much improvement.*
**Step 3:**
- If only the mean score of DASS is statistically higher than those of the assessment 1
→Follow the Distress protocol.
- Otherwise:
→Arrange the participant to a more intensive and individual psychotherapy provided by Psychology Department OR if the problem(s) can be solved by the primary researcher/assistant researcher, offer treatment to the participant with her/his consent.
→Withdraw the participant from the study and follow-up the participant's treatment.
→Report to Human Research Ethics Committee at ANU and Children's Hospital 1 and the supervisor.

**Distress Protocol**

Step 1: The participant informs that he/she is experiencing emotional distress.
Step 2: The primary investigator or assistant researcher contacts as soon as possible to assess his/her mental status via telephone or Facebook messenger, ask the participant:
- What are you feeling now?
- What are you thinking now?
- Are there anything else you want to tell me?
→Listen carefully and non-judgmentally. Try to explore as many details as possible. When he/she finishes telling, then wait a couple of minutes and ask:
- Are you ok now? Do you need more psychological support?
Step 3:
- The participant feels better, becomes calm, and does not need more support.
→Ask the participant: “Do you want to continue to participate in or withdraw from the study? ”, then response according to the reply.
- The participant is still distressed.
→Encourage the participant to contact psychological support services that were provided in Participation Information/Debrief Form OR if the problem(s) can be solved by the primary researcher/assistant researcher, offer treatment to the participant with her/his consent.
→Withdraw the participant from the study and follow-up the participant's treatment.
Report to Human Research Ethics Committee at ANU and Children's Hospital 1 and the supervisor.
